# F182. SYMPTOM-RELATED STRUCTURAL BRAIN PATTERN IN PATIENTS WITH SCHIZOPHRENIA-A PARTIAL LEAST SQUARE ANALYSIS

**DOI:** 10.1093/schbul/sby017.713

**Published:** 2018-04-01

**Authors:** Susanna Muckenhuber-Sternbauer, Anne Ruef, Peter Falkai, Dominic Dwyer, Nikolaos Koutsouleris

**Affiliations:** 1 Ludwig-Maximilians-University, University Psychiatric Hospital; 2 Ludwig-Maximilians-University

## Abstract

**Background:**

Multivariate neuroimaging studies of schizophrenia have revealed a generalizable neuroanatomical signature of the illness which however does not fully explain the variance of ist clinical phenotyps. A potential strategy to improve the mapping between the psychopathology and brain pathology of the disorder is to decipher the dictionary of symptom pattern and their neuroanatomical fingerprints. If successful, such a strategy could support a biologically informed revision of the taxonomy of psychosis.

**Methods:**

176 patients with first episode to chronic stages of schizophrenia were assessed using the Positive and Negative Syndrome Scale (PANSS) and scanned using T1-weighted magnetic resonance imaging (MRI). The patients`PANSS scores, sociodemographic data and disease course variables, as well as their grey matter volume maps (GMV) entered a multivariate Partial Least Square (PLS) analysis that decomposed unique patterns of brain-behavior covariance between these data domains into latent variales (LV). We tested the LVs for significance using nonparametric- permutation and bootstrap resampling techniques.

**Results:**

Three LVs showed significant brain-behavioral constellations. The first pattern linked hippocampal and medial frontal cortex volume with negative symptoms, age and age of onset. The second pattern consisted of opposite correlation between positive and negative symptoms associated with positive loadings in the subcortical structures such as the thalamus, the caudate nucleus and negative loadings in the auditory, insular and medial prefrontal cortices. The third LV presented a pattern involving negative symptoms, illness duration and age of onset as well as volume reductions in the anterior insular and orbitofrotal cortices.

**Discussion:**

Our results indicate that the heterogeneity of schizophrenia can be decomposed into clinically relevant brain-behavioral phenotyps of the disorder, suggesting a biologically-informed and disease stage-sensitive stratification of schizophrenic patients. This might provide a neurobiological basis for future stratified investigations of treatment effects and prognosis both in early and advanced stages of schizophrenia.

